# Modelling the sensitivity of life history traits to climate change in a temporary pool crustacean

**DOI:** 10.1038/srep29451

**Published:** 2016-07-11

**Authors:** Tom Pinceel, Bram Vanschoenwinkel, Luc Brendonck, Falko Buschke

**Affiliations:** 1 Laboratory of Aquatic Ecology, Evolution and Conservation, KU Leuven, Ch. Deberiotstraat 32, 3000 Leuven, Belgium; 2 Centre for Environmental Management, UFS, P.O. Box 339, Bloemfontein, 9300 South Africa; 3 Department of Biology, VUB, Pleinlaan 2, 1050 Brussels, Belgium

## Abstract

Temporary pool inhabitants face altered inundation regimes under climate change. While their exposure to these changes has received considerable attention, few studies have investigated their sensitivity or adaptability. Here, we use zooplankton as a model to explore how decreasing hydroperiods affect extinction risks and assess whether changes in life history traits could promote persistence. For this, we construct a three-stage matrix population model parameterised with realistic life-history values for the fairy shrimp *Branchipodopsis wolfi* from pools with varying hydroperiods. Our results suggest that extinction risks increase drastically once the median hydroperiod drops below a critical threshold. Although changes in life-history parameters could potentially compensate for this risk, the relative importance of each trait for population growth depends on the median hydroperiod. For example, survival of dormant eggs seemed to be most important when hydroperiods were short while the survival of freshly laid eggs and adult individuals were more important in longer-lived pools. Overall, this study demonstrates that zooplankton species are sensitive to climate change and that the adaptive capacity of organisms from temporary pools with dissimilar hydrology hinges on selection of different life history traits.

Climate change is expected to drive a rapid increase in species extinction rates^1^. Therefore, vulnerability assessment of organisms is needed and should integrate measures of their exposure, sensitivity and adaptability to predicted change^2^. In terms of exposure, temporary pool species are particularly vulnerable to the effects of climate change because shortening hydroperiods (i.e. length of the wet phase) are expected to impose more stringent time constraints on them to reach maturation and reproduce successfully^3^,^4^,^5^. Yet, the degree to which shortening hydroperiods affect population demographics remains understudied. Moreover, it is unclear whether changes in life history traits could allow populations to adapt to decreasing hydroperiods^6^.

Temporary pool zooplankton species are typically ‘r-selected’ with rapid maturation and high fecundity at a potential cost of life span^7^,^8^. Most populations rely on the production of dormant eggs to endure dry phases. These are added to an egg bank in the sediment from which a new active population hatches during a next inundation^9^. Egg banks may be characterised by partial hatching, which ensures that offspring is spread over several inundations and could be part of a bet hedging strategy that buffers populations against occasional reproductive catastrophes^10^. Theoretically, the hatching fraction of eggs is an important determinant of long term population fitness, especially under climate change^11^,^12^,^13^. However, its significance can only be interpreted in the context of other life-history traits, such as maturation time, fecundity and both egg- and adult survival.

In this study, we set out to quantify the sensitivity of zooplankton populations from temporary pools to changes in hydroperiod to obtain improved estimates of their vulnerability to climate change. For this, we construct a matrix population model to simulate population growth rates of the fairy shrimp model species *Branchipodopsis wolfi* (Crustacea, Branchiopoda, Anostraca) from rain fed rock pool habitats in South Africa. In addition, we examine the relative effect of changes in different life-history traits on population growth and extinction risk to identify those traits that are most likely to be under selection and that are key to the adaptive capacity of the species.

A major limitation of most models that attempt to link life history strategies to population growth under varying environmental quality is that they consider just two potential outcomes for each growing season: successful or non-successful reproduction^11^. In reality, variation among seasons is typically continuous. It is, however, often difficult to define an appropriate function that directly links the environment to reproductive success. Instead, some models draw reproduction stochastically for each growing season from a set of estimates^13^. The advantage of our model species is that active populations are strictly dependent on the presence of water and that we are able to directly link inundation length to reproductive output, as supported by empirical data^5^,^7^. This presents a conceptual extension of an earlier model by Ripley and co-workers^14^ for Californian fairy shrimp, in which reproductive output among growing seasons was drawn randomly from a set of likely values. Furthermore, analogous to a recent seed bank model for desert annuals^13^, we account for age-specific life-history trait values.

The direct link between reproduction and hydroperiod, which can be reconstructed for our study system, is a necessary step to investigate the potential consequences of different climate change scenarios on temporary pool biota. First of all, we predict that our simulations will demonstrate that a decrease in median hydroperiod negatively affects long term growth rates and increases the probability of population extinction. Second, we expect that changes in life-history parameters can compensate for these negative effects. Finally, we anticipate that the relative importance of different life-history parameters for long term population growth will depend on the hydroperiod.

## Results and Discussion

We investigated potential effects of decreasing hydroperiods on population growth rate and extinction probability of a fairy shrimp model species by constructing a matrix population model that was parameterised with realistic life-history values that were based on empirical data. Previous studies have shown that temporary pool zooplankton will face decreasing hydroperiods under climate change^3^,^4^,^5^ and our simulations confirm that these changes could threaten population persistence, despite the presence of an egg bank.

**Figure 1:**
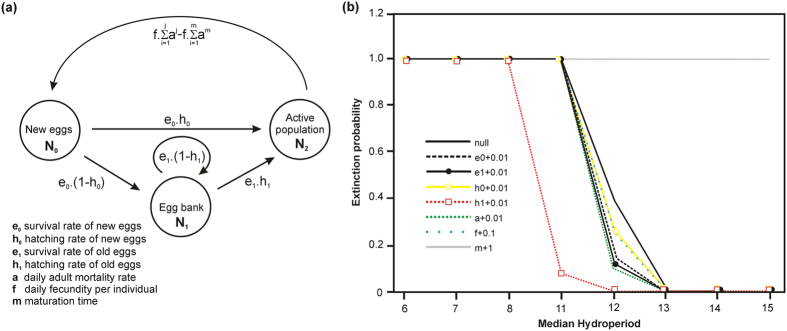
(**a**) Summary of *Branchipodopsis wolfi* population dynamics with reproduction. The matrix population model comprises three stage classes; eggs produced during the previous inundation(N_0_), older eggs(N_1_) and the active population(N_2_). (**b**) Impact of small changes in life history trait values on the probability of population extinction under different median hydroperiods.

Simulations indicate that *B. wolfi* can only maintain populations in temporary pools with a minimum median hydroperiod of 12 days (Fig. 1b). The extinction probability increased from ±0.6% to ±100% when the median hydroperiod decreased from 13 to 11 days in our simulations. These non-linear dynamics of extinction risk are worrying because global warming will likely reduce the average and median hydroperiod of the studied habitats by as much as 15% and 29–41%, respectively (K. Tuytens unpublished data^5^), which could result in local extinction if populations cannot be replenished by immigration from other pools.

**Figure 2:**
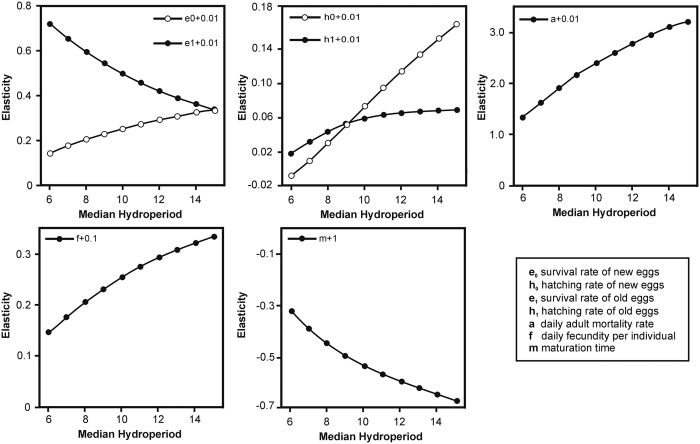
Elasticity of long-term growth rates of *Branchipodopsis wolfi* populations under small changes in life history parameters and different median hydroperiods.

However, the results from our study suggest that small changes in life-history traits at least partly compensate for the increased extinction probability under reduced median hydroperiods (Fig. 1b). Increasing trait values for adult or egg survival, fecundity and hatching fraction generally lowered the probability of population extinction for a given median hydroperiod. The only exception was age at sexual maturation, for which a one day increase in maturation time ensured that the egg bank was depleted and the population always went extinct in pools with a median hydroperiod shorter than 15 days. Changes in adult survival had the largest impact. Even an increase in daily adult survival rate by just 1% lowered the extinction probability by ±40–80%. Consistent with this result, our elasticity analysis suggested a strong effect of changes in adult survival on long term population growth, especially in long lived pools (Fig. 2). This finding demonstrates the importance of accounting for biotic interactions when assessing vulnerability under climate change since predation and competition can have a major influence on zooplankton survival rates in temporary pools^15^. It must be noted that while we explored the impact of changes in life history traits on population growth and extinction probability, we did not distinguish between different processes that could lead to such changes. For instance, changes in life history trait values could directly result from exposure to increased temperatures under climate change or be brought about through phenotypic plasticity or (epi-)genetic change^2^,^6^.

The current model only assesses indirect effects of temperature change on population growth and persistence (i.e. temperature change as a driver of variation in hydroperiod), but not direct effects on life history traits. It is likely that climate change in southern Africa will alter seasonal rainfall patterns, resulting in completely novel combinations of temperature and precipitation^16^. As such, it is difficult to predict how temperature will be correlated to hydroperiod. However, it is possible to speculate that changing temperature will affect life history traits in ways that would be tractable in our model. For instance, maturation rates could increase with temperature. Nonetheless, making quantitative links between temperature and life history traits requires further experiments to identify temperature response curves and physiological thresholds. Therefore, we chose to simply modify the life history traits directly without explicitly considering a potential temperature-dependency.

Our simulations suggest variable contributions of different life-history traits to population growth along the pond permanence gradient. While sensitivity and elasticity analysis showed that the survival rate of new eggs increases in importance in long-lived pools, the positive effect of survival of older eggs from the egg bank increases as hydroperiods decrease (Fig. 2, Fig. S3). This implies that the egg bank plays a more prominent role for population persistence in short-lived compared to long-lived pools, matching theoretical predictions^9^,^10^,^11^. Short-lived pools typically also have higher levels of habitat uncertainty^5^, driving selection for lower hatching fractions as part of a bet hedging strategy^12^ and increasing the importance of survival in the egg bank^10^.

If short-term population sizes fluctuate drastically, even populations with positive long-term growth rates may have non-zero extinction risks^17^,^18^. Yet, our study suggests that such strong fluctuations may in fact increase persistence if rare long inundations replenish the egg bank sufficiently to offset the more common (but smaller) decreases in population size caused by short inundations (Supplementary results and Discussion; Fig. S1). This could be an example of antifragility in an ecological system, where the system actually benefits from volatility^19^.

Overall, we can conclude that the vulnerability of temporary pool zooplankton to climate change should be assessed using an integrated approach that transcends simple measures of exposure and incorporates studies of species sensitivity and adaptive capacity. For instance, the sensitivity of population growth rate to adult survival highlights the importance of experiments that include competitive and predatory interactions. Therefore, experimental evolution trials under realistic conditions should be the next step towards confirming the patterns and predictions revealed by our simple model.

## Methods

All *B. wolfi* life-history parameters were derived from published sources^20^,^21^,^22^,^23^ or best estimates from unpublished lab and field observations (Table 1). The matrix population model comprises three stage classes to account for age-specific trait values; (1) eggs that were produced during the previous inundation (N_0_), (2) older eggs in the egg bank (N_1_) and (3) active populations (N_2_) (Fig. 1a). We made two assumptions to keep the model as generic and simple as possible; (1) a fraction of eggs hatches at the onset of any inundation and (2) life history parameters are constant and do not exhibit density dependence (Supplementary methods S1).

We ran all simulations in R version 3.12 (R Core Team, 2014) with starting values of N_0_ = N_2_ = 0 and N_1_ = 25000. To capture the effects of hydroperiod stochasticity on population dynamics, we iterated our model 1000 times and based our conclusions on the average of these iterations. Each iteration ran over a time-series of 1100 inundations and hydroperiod (j) sequences were simulated to approximate those found in nature (Supplementary methods S2; Fig. S2)^5^. We calculated the stochastic population growth rate (logλ) of the number of eggs in the egg bank (N_1_) and the variance (σ^2^) of N_1_ across successive inundations^17^. If the age at sexual maturation (m) of the fairy shrimps was lower than the hydroperiod (m < j), we modelled these changes as (1):


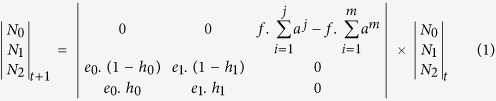


We differentiated between the egg survival (e) and hatching (h) rates of N_0_ (e_0_ and h_0_, respectively) and N_1_ (e_1_ and h_1_, respectively). For N_2_, daily fecundity (f) was averaged per breeding pair since the fairy shrimps only reproduce sexually. Therefore, N_0_ for any given inundation was the sum of the total fecundity on each day, considering that only a proportion of adults (a) survived to the next day. If the active population could not reach sexual maturity (m > j), population dynamics were modelled as (2):


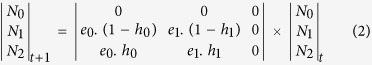


Finally, to assess the effect of changes in life-history parameters on long-term stochastic population growth rates, we performed sensitivity and elasticity analyses (Supplementary methods S3). The sensitivity analysis quantified the change in population growth rates by sequentially adjusting each life-history parameter by suitably small increments. However, such a sensitivity analysis is often difficult to interpret when the life-history parameters are in differently scaled units (e.g. proportional survival rates compared to maturation time in days). The elasticity analysis, therefore, represents the effects of incremental changes in life-history parameters on population growth rates while simultaneously accounting for the variable scaling of these parameters.

When long term population growth rates are negative, the probability of extinction is 1. However, if the variance in short-term changes in population size is high, extinction is possible even with positive growth rates. Following Caswell^17^, we therefore estimated the extinction probability (E) as (3):


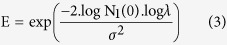


where N_1_(0) is the starting number of dormant eggs in the egg bank. Subsequently, we assessed the sensitivity of E to changes in the different life-history parameters by sequentially increasing each of them while keeping the others constant (Supplementary methods S3).

## Additional Information

**How to cite this article**: Pinceel, T. *et al*. Modelling the sensitivity of life history traits to climate change in a temporary pool crustacean. *Sci. Rep*. **6**, 29451; doi: 10.1038/srep29451 (2016).

## Supplementary Material

Supplementary Information

## Figures and Tables

**Table 1:** *Branchipodopsis wolfi* life-history parameters were obtained from the literature, field and laboratory experiments.

Vital rate	Mean	Range	Source
Egg survival	e_0_*: 55%	e_0_*: 10–80%	22
	e_1_*: 80%	e_1_*: 50–90%	22
Adult survival(a)	a: 74%	a: 60–99%	Unpublished data; L. Brendonck field observations
Hatching fraction(h)	h_0_: 47%	h1: 21–67%	22, 23, Unpublished data
	h_1_: 9%	h2: 0–57%	22, Unpublished data
Maturation time (m)	m: 6	m: 5–8	20, 21, 23
Daily fecundity (f)	f: 13	f: 12–17	20, 21

^*^This represents the best possible estimate rather than an exact value.
