# Supplementary material for: Modelling the sensitivity of life history traits to climate change in a temporary pool crustacean
Source: Sci Rep. 2016 Jul 11;6:29451. doi: 10.1038/srep29451 (PMC4941417; doi:10.1038/srep29451)
Supplement: Supplementary Information [file srep29451-s1.pdf]

Modelling the sensitivity of life history traits to climate change in a temporary pool crustacean

Tom Pinceel[1, 3*], Bram Vanschoenwinkel[2], Luc Brendonck[1] & Falko Buschke[3]

[1]Laboratory of Aquatic Ecology, Evolution and Conservation, KU Leuven, Ch. Deberiotstraat 32, 3000 Leuven, Belgium

[2] Department of Biology, VUB, Pleinlaan 2, 1050 Brussels, Belgium

[3]Centre for Environmental Management, UFS, P.O. Box 339, Bloemfontein, 9300 South Africa

*E-mail: tom.pinceel@bio.kuleuven.be

Supplementary Results & Discussion

Contrasting short- and long-term growth rates

Fairy shrimp populations in stochastic environments are typically characterised by high variance in short-term population growth rates. This means that, even if populations exhibit positive growth rates over an extended period of time, they might show dramatic declines in population sizes in the short-term. To illustrate this, we examined the short-term changes in population size for one iteration of our model for a pool with a median hydroperiod of 12 days. We specifically selected this hydroperiod because it appears to represent the threshold needed for positive long-term population growth rate under the life history parameters in our simulations. Indeed, the random iteration we examined showed a positive population growth rate of 3.18 % ($\log\lambda$ = 1.0318), despite the fact that the population only increased during 42.2 % of all inundations. During 30.5 % of all inundations the fairy shrimp actually reached sexual maturity and produced eggs. Yet, these increases in numbers were not sufficient to offset natural mortality rates (Figure S3). The long-tail of short-term changes in population size illustrates that positive population growth rates require only a minority of inundations that contribute disproportionaty to the overall population.

This long-tailed pattern is consistient with the concept of antifragility, in which the dynamics of a system are disproportionally influenced by a few rare events (the so-called 'black swan' events). Antifragile systems – in contrast to fragile ones – tend to benefit from volatility in the form of rare events; in this case, relatively rare inundations in which populations can potentially double in size. This is the ecological antithesis of extinction caused by rare 'disaster' events (Taleb, 2012).

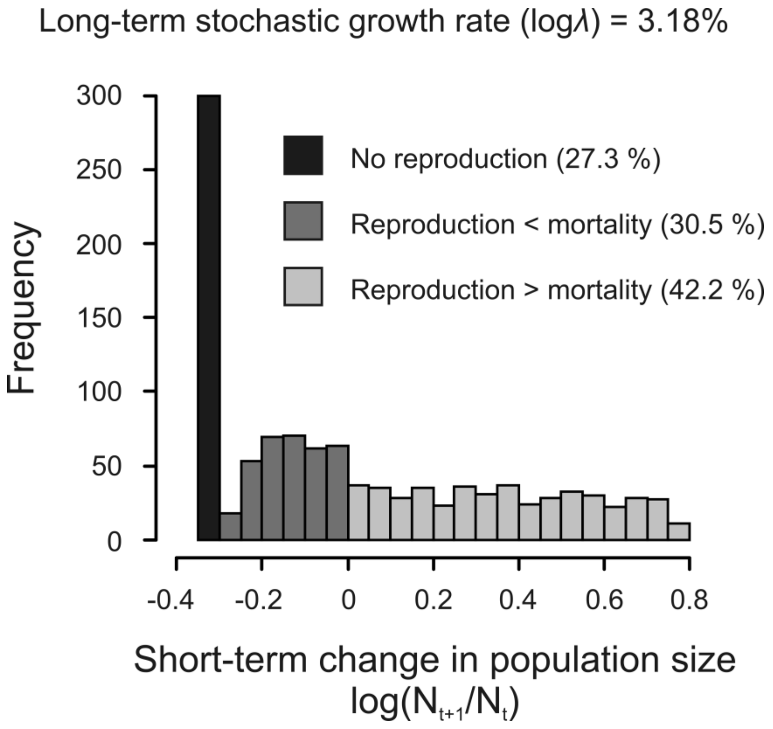

Figure S1 Short-term changes in population size for a single iteration of our model demonstrate that positive long-term population growth rate is possible, even though the population declines during the majority of inundations, if a minority of inundations contributes disproportionately to the overall population.

Supplementary methods

S1: ***Model assumptions***

We specifically opted for a basic model that allows us to explore general principles as a starting point to test more complex hypotheses. It was not our aim to build a realistic egg bank budget model for individual populations. Therefore, we simplified the model by making two assumptions.

First of all, it must be stressed that the current model assumes no density dependence of life history parameters and does not include biotic interactions with other trophic levels or spatial rescue effects. The 'r-strategy' (boom and bust) of fairy shrimps, which emerge during the beginning of inundations when food is plentiful, could plead against strong density dependent growth. Yet, the very high mortality rates among juvenile shrimps, during early stages of the inundation, that have been observed in the field could hint at competition for food (B. Vanschoenwinkel, unpublished data). In reality, competitive and predatory interactions are likely to change due to climate change. Interacting species could be differentially affected by the changing environment, which could increase or decrease the strength of interspecific interactions promoting or decreasing persistence.

Second, we did not take into account the fact that eggs could respond adequately to hatching cues which would allow them to avoid part of the unsuitable inundations. In this respect, both dispersal and phenotypic plasticity could also buffer against extinction in the studied systems (Vanschoenwinkel et al., 2013). For instance, *B. wolfi* eggs have been shown to refrain from hatching under elevated conductivities (<125µs cm^{-1}) which could represent an adaptation to avoid reproductive catastrophes since high conductivities signal high probabilities of early drying (Vanschoenwinkel et al., 2010). In addition, hatching of *B. wolfi* eggs is only initiated in the presence of light. This could serve to avoid hatching under thick layers of sediment where emerged larvae would be trapped (Pinceel et al., 2013). Therefore, the long term population growth rates discussed in this study are simplistic and changes in response to specific parameters should be interpreted in a relative rather than in an absolute way.

S2: ***Log-normal approximation for hydroperiod***

Since we specifically sought to capture the effects of hydroperiod stochasticity on population dynamics, we iterated our model 1000 times and based our conclusions on the average of these iterations. For each iteration, we first simulated the sequence of hydroperiods by randomly drawing 1100 values from a log-normal distribution where the log-mean represented the median inundation length in days and the log-standard deviation was 1. This convenient approximation allowed us to easily simulate continuous changes in hydroperiod. This resulted in a sequence of 1100 independent inundations of which most were short (<3 days); closely corresponding to the frequency distribution of hydroperiods for mechanistically simulated inundations of real rock pools on the Korannaberg inselberg in South Africa (Vanschoenwinkel et al. 2009; Tuytens et al. 2014). Those mechanistic simulations incorporated empirical data on rainfall, evaporation, inflow and overflow and were calibrated using pool-specific catchment factors estimated from field data.

It would have been possible to incorporate these empirically-supported hydroperiods in our simulations. We chose, however, to use the log-normal approximation for two reasons. First of all, the simple approximation allowed us to change the median hydroperiod incrementally without constraints. Second, the approximation allowed us to standardise the length of the time-series (1100), regardless of the median hydroperiod. The more-realistic hydroperiod data suffers in that the rock pools in nature do not necessarily vary in hydroperiod across a continuous gradient. Moreover, long-lived pools with long hydroperiods tend to have fewer separate inundations than short-lived pools for a specific interval (say, the interval of available climate data), which makes standardised comparisons difficult. Nevertheless, our log-normal approximation seems to capture natural variation in hydroperiod across a range of median inundation lengths (Figure S1).

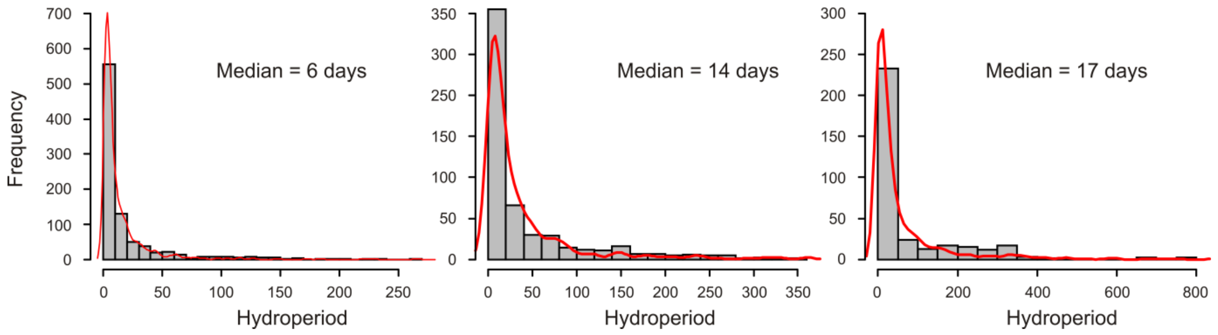

Figure S2: The log-normal approximation of hydroperiod (red line) and data generated from specifically-calibrated mechanistic models (grey bars) for pools with median inundation lengths of 6, 14 and 17 days are highly similar.

S3: ***Stochastic population growth rate***

The main text – and the references therein – describe the matrices used in our simulations of population dynamics. Here, we elaborate on the procedures for determining stochastic population growth rates as well as for the sensitivity and elasticity analyses in more detail.

For each iteration of our model, we simulated the population size of dormant eggs in the egg bank (N_1) for every one of the 1100 time-steps (t). After allowing a burn-in period of 100 time-steps to account for transient dynamics based on the starting conditions, we calculated the stochastic population growth rate (logλ) as:

$$\log \lambda = \frac{1}{1000} \sum_{t=101}^{1100} \log\left(\frac{N_{1(t+1)}}{N_{1(t)}} \right)$$

Sensitivity and elasticity analyses

Sensitivity analysis (Fig. S3) is a way to quantify the effect of 'small' changes (s) in one of the life history parameters on stochastic population growth rate. This was done by manually increasing and decreasing life history parameters, one at a time, in small increments and quantifying the impact of these changes on growth rates. Specifically, all demographic parameters, expressed as proportions (e_0, e_1, h_0, h_1 and a), were increased by 0.01 whereas the non-proportional parameters fecundity and maturation time were increased by 0.1 and 1, respectively. Sensitivity (Fig. S3) can, therefore, be quantified as:

$$\frac{\lambda * - \lambda}{s}$$

Here, λ* and λ are the exponent of the stochastic population growth rates after and before, respectively, the incremental change in the life history parameter.

However, incremental changes in life history parameters might have differing effects on growth rates when these parameters are in different units (e.g. proportional survival rates compared to maturation ages in days). Elasticity (main text Fig. 2) is a way to correct for the scaling of parameters when comparing growth rates:

$$\frac{sensitivity \times \{parameter\}}{\lambda}$$

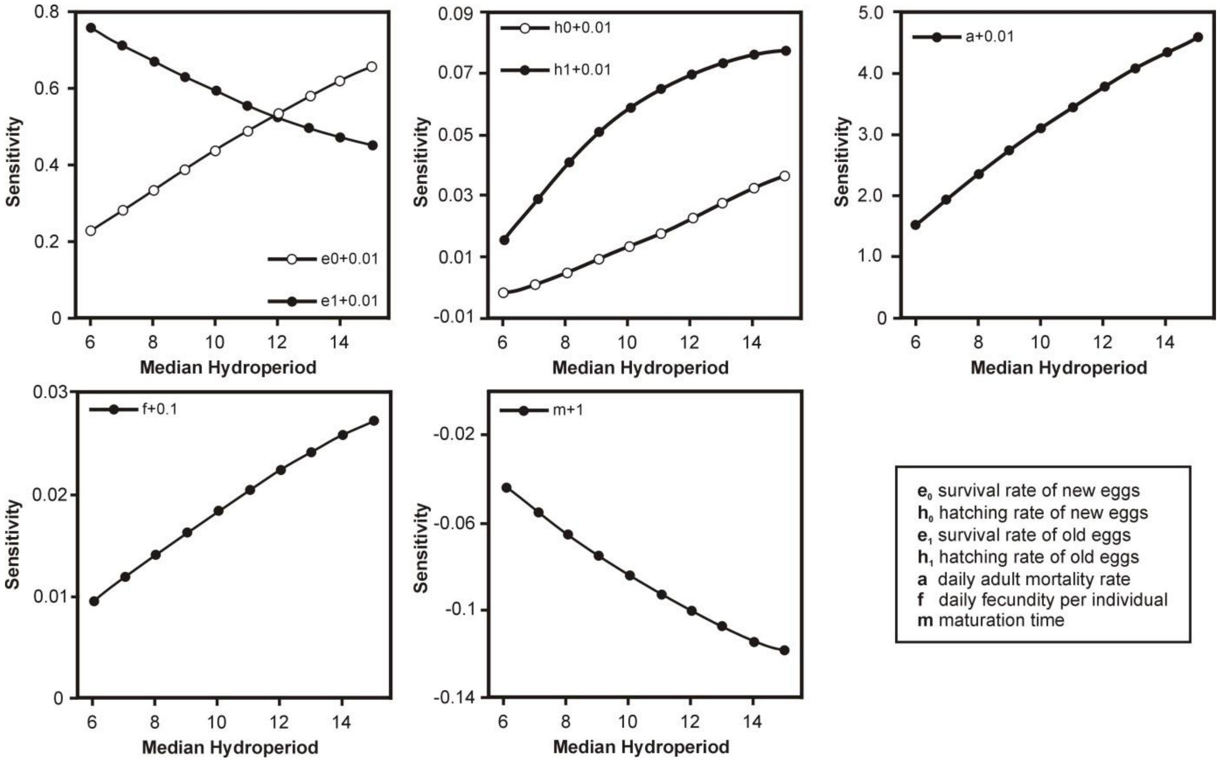

Figure S3 The sensitivity of the long-term population growth rate of *B. wolfi* populations to small changes in life history parameters for different median hydroperiods.

References Supplementary information

Pinceel T, Vanschoenwinkel B, Uten J ,Brendonck L. 2013 Mechanistic and evolutionary aspects of light-induced dormancy termination in a temporary pond crustacean. *Freshw. Sci.* **32**, 517-524. (doi:10.1899/12.157.1).

Taleb NN. 2012 Anti-fragile: How to Live in a World We D

on't Understand, Allen Lane.

Tuytens K, Vanschoenwinkel B, Waterkeyn A ,Brendonck L. 2014 Predictions of climate change infer increased environmental harshness and altered connectivity in a cluster of temporary pools. *Freshw. Biol.* **59**, 955-968. (doi:10.1111/fwb.12319).

Vanschoenwinkel B, Hulsmans A, De Roeck ER, De Vries C, Seaman M ,Brendonck L. 2009 Community structure in temporary freshwater pools: disentangling effects of habitat size and hydroregime *Freshw. Biol.* **54**, 1487-1500. (doi:10.1111/j.1365-2427.2009.02198).

Vanschoenwinkel B, Seaman MT ,Brendonck L. 2010 Hatching phenology, life history and egg bank size of a fairy shrimp (Branchiopoda, Crustacea) in relation to the ephemerality of its habitat. *Aquatic Ecol.* **44**, 771-780. (doi:10.1007/s10452-010-9315-y).

Vanschoenwinkel B, Buschke F ,Brendonck L. 2013 Disturbance regime alters the impact of dispersal on alpha and beta diversity in a natural metacommunity. *Ecology* **94**, 2547-2557. (doi:10.1890/12-1576.1).
